# Effectiveness of COVID-19 Vaccines against Delta (B.1.617.2) Variant: A Systematic Review and Meta-Analysis of Clinical Studies

**DOI:** 10.3390/vaccines10010023

**Published:** 2021-12-25

**Authors:** Ali Pormohammad, Mohammad Zarei, Saied Ghorbani, Mehdi Mohammadi, Saeideh Aghayari Sheikh Neshin, Alireza Khatami, Diana L. Turner, Shirin Djalalinia, Seied Asadollah Mousavi, Heydar Ali Mardani-Fard, Amir Kasaeian, Raymond J. Turner

**Affiliations:** 1 Department of Biological Sciences, University of Calgary, Calgary, AB T2N 1N4, Canada; ali.pormohammad@ucalgary.ca (A.P.); mehdi.mohammadiashan@ucalgary.ca (M.M.); 2 Renal Division, Brigham & Women’s Hospital, Harvard Medical School, Boston, MA 02115, USA; mzarei@hsph.harvard.edu; 3 John B. Little Center for Radiation Sciences, Harvard T.H. Chan School of Public Health, Boston, MA 02115, USA; 4 Department of Virology, Faculty of Medicine, Iran University of Medical Science, Tehran 1449614535, Iran; ghorbani.sai@iums.ac.ir (S.G.); khatami@iums.ac.ir (A.K.); 5 Neuroscience Research Center, Guilan University of Medical Sciences, Rasht 4188794755, Iran; saeidehyari88@gmail.com; 6 Department of Family Medicine, Cumming School of Medicine, University of Calgary, Calgary, AB T2N 4N1, Canada; diana.turner@albertahealthservices.ca; 7 Deputy of Research and Technology, Ministry of Health and Medical Education, Tehran 1467664961, Iran; sh_djalalinia@alumni.tums.ac.ir; 8 Non-Communicable Diseases Research Center, Endocrinology and Metabolism Population Sciences Institute, Tehran University of Medical Sciences, Tehran 1411713139, Iran; 9 Hematology, Oncology and Stem Cell Transplantation Research Center, Research Institute for Oncology, Hematology and Cell Therapy, Tehran University of Medical Sciences, Tehran 1411713131, Iran; a_mousavi@tums.ac.ir; 10 Department of Mathematics, Yasouj University, Yasouj 7493475918, Iran; h_mardanifard@yu.ac.ir; 11 Digestive Diseases Research Center, Digestive Diseases Research Institute, Tehran University of Medical Sciences, Tehran 1411713135, Iran; 12 Inflammation Research Center, Tehran University of Medical Sciences, Tehran 1411713137, Iran

**Keywords:** COVID-19, SARS-CoV-2, SARS-CoV-2 variants, SARS-CoV-2 B.1.617.2 variant vaccines, Delta variant, efficacy, side effect, meta-analysis, effectiveness

## Abstract

The high transmissibility, mortality, and morbidity rate of the SARS-CoV-2 Delta (B.1.617.2) variant have raised concerns regarding vaccine effectiveness (VE). To address this issue, all publications relevant to the effectiveness of vaccines against the Delta variant were searched in the Web of Science, Scopus, EMBASE, and Medline (via PubMed) databases up to 15 October 2021. A total of 15 studies (36 datasets) were included in the meta-analysis. After the first dose, the VE against the Delta variant for each vaccine was 0.567 (95% CI 0.520–0.613) for Pfizer-BioNTech, 0.72 (95% CI 0.589–0.822) for Moderna, 0.44 (95% CI 0.301–0.588) for AstraZeneca, and 0.138 (95% CI 0.076–0.237) for CoronaVac. Meta-analysis of 2,375,957 vaccinated cases showed that the Pfizer-BioNTech vaccine had the highest VE against the infection after the second dose, at 0.837 (95% CI 0.672–0.928), and third dose, at 0.972 (95% CI 0.96–0.978), as well as the highest VE for the prevention of severe infection or death, at 0.985 (95% CI 0.95–0.99), amongst all COVID-19 vaccines. The short-term effectiveness of vaccines, especially mRNA-based vaccines, for the prevention of the Delta variant infection, hospitalization, severe infection, and death is supported by this study. Limitations include a lack of long-term efficacy data, and under-reporting of COVID-19 infection cases in observational studies, which has the potential to falsely skew VE rates. Overall, this study supports the decisions by public health decision makers to promote the population vaccination rate to control the Delta variant infection and the emergence of further variants.

## 1. Introduction

Severe acute respiratory syndrome coronavirus 2 (SARS-CoV-2) caused more than 82 million known cases by the end of 2020 [1]. To prevent the spread of COVID-19, significant progress has been achieved in developing several COVID-19 vaccines to reduce the rate of transmission, severity of the disease, symptom development, and hospitalizations and increase the recovery rate [2]. This has been demonstrated by clinical trials [3,4,5] and real-world evidence [6,7,8,9]. However, the emergence of new variants, especially those related to the spike gene function, threatens the efficacy of current vaccines [10]. New variants that acquire genomic mutations leading to enhanced transmission, immune system evasion, and/or pathogenicity have been classified as Variants of Concern (VOCs). The World Health Organization (WHO) reported the main SARS-CoV-2 VOCs along with the country of the first detection and the date of designation including the Alpha variant (B.1.1.7) in the United Kingdom on 18 December 2020, the Beta variant (B.1.351) in South Africa on 18 December 2020, the Gamma variant (P.1) in Brazil on 11 January 2021, the Delta variant (B.1.617.2) in India on 11 May 2021 [11], and the most recent variant, Omicron (B.1.1.529), in South Africa on 26 November 2021 [11,12,13].

The spike (S) protein, which is the principal target in COVID-19 vaccines [13], is one of the five structural proteins of SARS-CoV-2 that has two different subunits. The S1 subunit, which contains the receptor-binding domain, mediates attachment to the cell surface receptors used by SARS-CoV-2, mainly the angiotensin-converting enzyme 2. The S2 subunit is responsible for the membrane fusion that is necessary for the viral entry into the host cell [14]. As with other RNA viruses, SARS-CoV-2 benefits from a high mutation rate, resulting in extensive adaptability [15]. Mutations in the receptor-binding domain of the S protein seem to have a crucial impact on the infectivity, immune reaction, and pathogenicity of the virus [16]. Since the new variants of SARS-CoV-2 have multiple mutations including those in the S protein, there is a concern whether the mutations might change the antigenicity of the virus and subsequently the effectiveness of vaccines [17].

The Delta variant, one of the VOCs, caused a significant surge in COVID-19 cases in India, reaching over 400,000 daily new cases and 4000 daily new deaths by May 2021 [18]. Moreover, this variant was reported in 43 countries simultaneously, including drastic surge cases in the United Kingdom, mostly linked with traveling from India and community transmission [19], which created concern since the effectiveness of the existing vaccines against this variant was unclear [20].

Recently, the new VOC, named Omicron by the WHO, has raised public fears, and as suggested by preliminary evidence, it has increased the risk of breakthrough infections [12]. The question of whether vaccination is effective against Omicron needs more data. Yet, Delta is the dominant variant in many countries [12]. To address the knowledge gap of vaccine effectiveness (VE) towards the SARS-CoV-2 Delta variant, we instigated a comprehensive data review. The VE of existing COVID-19 vaccines, including two mRNA-based vaccines, Pfizer-BioNTech and Moderna, one viral vector vaccine, AstraZeneca, and two inactivated virus vaccines, Bharat Biotech and CoronaVac, was comprehensively explored based on available published data on the Delta variant after the first, second, and third doses of vaccination. Additionally, the effectiveness of each vaccine in preventing infection, hospitalization, and severe infection or death was covered, thus providing important data regarding the effectiveness of vaccines against the Delta variant.

## 2. Methods

### 2.1. Search Strategy

This analysis followed the recommendations of the priority report items in the Systematic Review and Meta-Analysis statement (PRISMA) [21]. We searched all publications related to the VE against the SARS-CoV-2 (B.1.617.2) Delta variant from the following databases: Web of Science, Scopus, EMBASE, and Medline (via PubMed). All studies were searched without language restriction by two independent reviewers up to 15 October 2021. Search Medical Subject Headings (MeSH) terms used were “Vaccine”, “SARS-CoV-2 Vaccine”, and “Delta variant” or “B.1.617.2” as well as all synonyms such as booster shot, boost immunity, and COVID-19 vaccine. We searched the unpublished gray literature using the Centers for Disease Control and Prevention (CDC), the World Health Organization (WHO), and the Google Scholar academic search engine. The selected articles and relevant review references and citation lists were also checked for other related references (forward and backward citations recommended by Cochrane). See Table S1 for more information on search strategies.

### 2.2. Study Selection

The records were first reviewed by three independent authors based on the title and abstract (SGh, AP, and AKh). All unrelated publications were removed, and the full texts of the remaining articles were reviewed. Two independent reviewers (AP and SGh) evaluated potentially appropriate articles, and discussions resolved disagreements until an agreement was reached on each article [22].

### 2.3. Eligibility and Inclusion Criteria

The following predefined conditions had to be met in order to be considered for inclusion in this meta-analysis: in the initial screening, all studies reporting the VE against the Delta (B.1.617.2) variant were included in the systematic review and meta-analysis.

### 2.4. Exclusion Criteria

Preclinical studies, fauna studies, meta-analyses, editorial letters, reviews, studies without extractable data, news reports, press releases, modeling studies, media reports, presentations, and studies on variants other than Delta as well as studies with syndromic outcomes without laboratory confirmation of the Delta variant were excluded from the meta-analysis.

### 2.5. Data Extraction

Three independent reviewers extracted data from selected studies. The following data were collected from each article: first author, year of publication, vaccine name, company, study type, vaccine type, country, number of cases, doses, days after vaccination, VE for Delta variant, hospitalization efficacy, and efficacy for serious illness or death. Three authors (SGh, AKh, and AP) independently extracted the data, and another author (MZ) randomly checked the extracted data. Any disagreement in the data was rechecked by all and resolved amicably. Studies that reported the VE after the first, second, and third doses were considered as different datasets.

### 2.6. Quality Assessment

The study quality assessment was conducted independently by two reviewers based on a revised assessment checklist recommended by the Joanna Briggs Institute [23], and the differences were resolved by consensus. The checklist consisted of nine questions that reviewers utilized for each study. The answer to each question if “Yes” received a score of 1, while “no” received a 0. Therefore, the endpoints for each study ranged from 0 to 9 (Table S2).

### 2.7. Analysis

All extracted data were initially cleaned and prepared for analysis via Microsoft Office 365, and analysis was performed by Comprehensive Meta-Analysis Software Version 2.0. The point estimates of the effect size and 95% confidence interval (95% CI) were calculated for estimating VE. The VE prediction in the included studies is explained in the Supplementary Materials. Random effects models were used to estimate pooled effects. Additionally, to search for heterogeneity between studies, the I2 statistic was used, and high heterogeneity was characterized as an I2 > 50%, with sources of heterogeneity established through meta-regression and subgroup analyses. For all analyses, two-tailed statistics and a significance level of less than 0.05 were considered. Begg’s test and Egger’s test were conducted for the detection of publication bias.

The test statistic is a z-score (z) defined by the following equation: z = (p − P)σ, where P is the hypothesized value of the population proportion in the null hypothesis, p is the sample proportion, and σ is the standard deviation of the sampling distribution [24]. For the null test, to achieve a significance level of 0.05 for a two-sided test, the absolute value of the test statistic (|z|) must be greater than or equal to the critical value 1.96. 

## 3. Result

### 3.1. Characteristics of Included Studies

**Figure 1:**
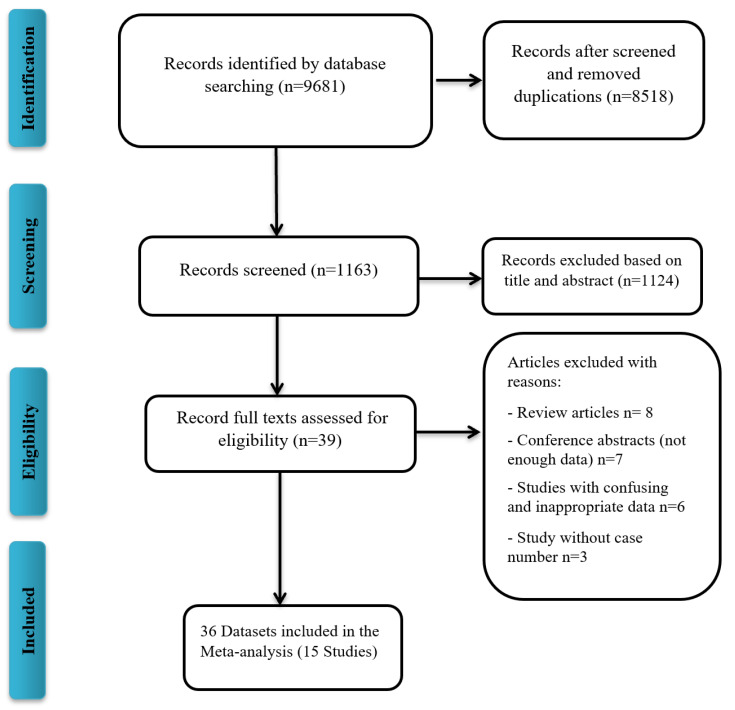
Flow diagram of literature search and study selection for meta-analysis (PRISMA flow chart).

A total of 9681 publications were screened for COVID-19 VE against the Delta variant, of which 15 studies, all written in English, were included in the meta-analysis (Figure 1). Characteristics of the included publications are summarized in Table 1. A total of 15 studies (36 datasets) were included in the meta-analysis. Studies that reported different vaccine dose efficacies were considered as a separate dataset for the meta-analysis. A total of 10 datasets reported the VE against the Delta variant after the first dose, 23 datasets after the second dose, and 3 datasets after the third dose. Out of 36 datasets, 16 datasets reported the Pfizer-BioNTech VE, 8 that of AstraZeneca, 5 that of Moderna, 2 that of mRNA vaccines (Pfizer/Moderna), 3 that of CoronaVac, 1 that of Pfizer/Moderna/J&J, and 1 that of Bharat Biotech VE. All included studies were reported during the 2021 publication year. Begg’s test (*p* = 0.37) and Egger’s test (*p* = 0.47) did not detect significant publication bias.

### 3.2. Efficacy of Different COVID-19 Vaccines against Delta Variant Infection 

#### 3.2.1. Pfizer-BioNTech Efficacy against COVID-19 Delta Variant Infection 

**Figure 2:**
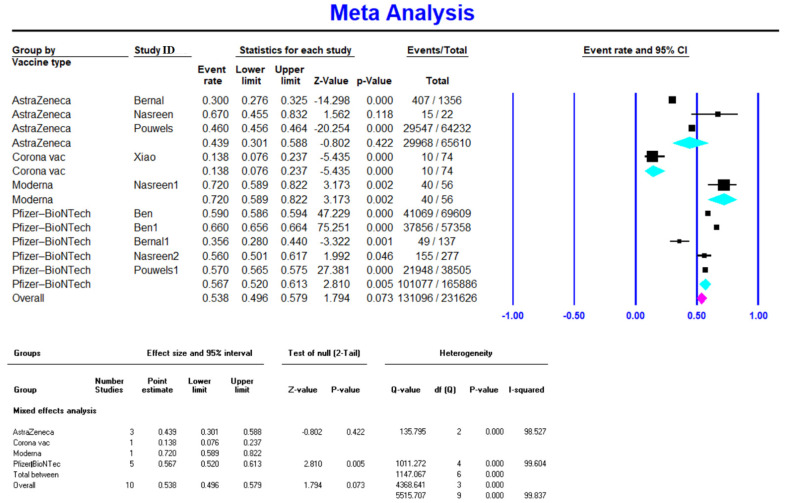
Effectiveness of COVID-19 vaccines after the first dose against Delta variant infection.

**Figure 3:**
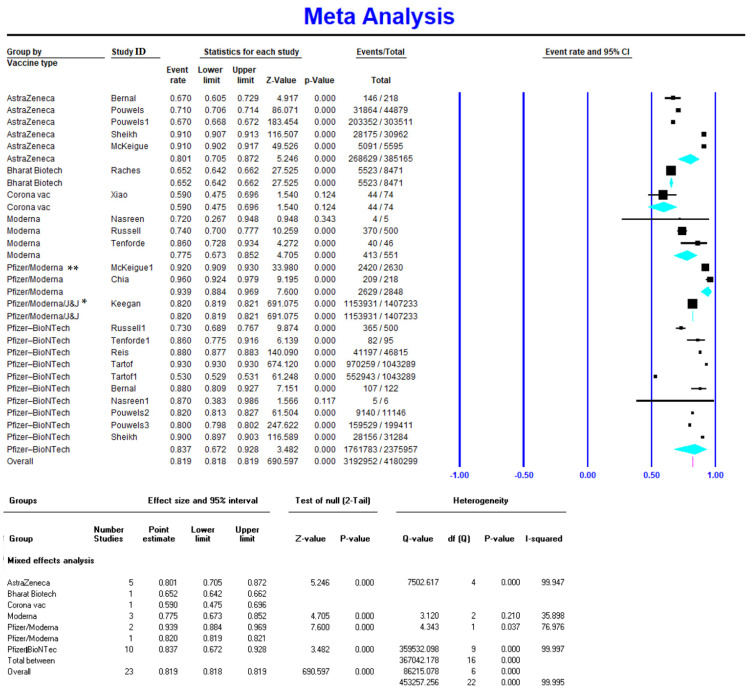
Effectiveness of COVID-19 vaccines after the second dose against Delta variant infection. * Reported for mRNA vaccines (Pfizer/Moderna); ** total of 52.9% included cases vaccinated with Pfizer, 38.1% with Moderna, and 9.05% with Janssen (Pfizer/Moderna/J&J).

Meta-analysis on five studies (165,886 vaccinated cases in total) showed a VE of 0.567 (95% CI 0.520–0.613) for the Pfizer-BioNTech vaccine after the first dose (Figure 2). Available data on 2,375,957 vaccinated individuals (10 studies) showed a VE of 0.837 (95% CI 0.672–0.928) for the Pfizer-BioNTech vaccine after the second dose (Figure 3). Similarly, Pfizer-BioNTech had a VE of 0.972 (95% CI 0.96–0.978) after the third vaccine dose (reported by one study) [18] (Table 2).

#### 3.2.2. Moderna Efficacy against COVID-19 Delta Variant Infection

Only one study with 56 vaccinated cases reported the VE of Moderna after the first dose, which had the highest first-dose VE amongst all vaccines at 0.72 (95% CI 0.589–0.822) [27]. Three studies (551 vaccinated cases in total) reported Moderna’s VE after the second dose at 0.775 (95% CI 0.673–0.852), and one study with 256 vaccinated cases reported a VE of 0.97 (95% CI 0.964–0.978) after the third dose [18].

#### 3.2.3. AstraZeneca Efficacy against COVID-19 Delta Variant Infection

AstraZeneca had the highest VE after the RNA-based vaccines (Pfizer-BioNTech and Moderna) against the Delta variant of COVID-19. It had a VE of 0.44 (95% CI 0.301–0.588) after the first dose [25,26,37] and 0.801 (95% CI 0.705–0.872) after the second dose [26,28,31,32]. Data were not available for the VE of AstraZeneca after the third dose.

#### 3.2.4. CoronaVac Efficacy against COVID-19 Delta Variant Infection

Only one study reported CoronaVac’s VE, which was 0.138 (95% CI 0.076–0.237) [29] after the first dose and 0.59 (95% CI 0.475–0.696) [27] after the second dose.

There are very limited reports for the VE of other vaccines against the Delta variant of COVID-19. Only one study reported the VE of Bharat Biotech, with a VE of 0.652 (95% CI 0.642–0.662) after the second dose [38], and one study reported a VE of 0.638 (95% CI 0.631–0.643) after the third dose for CoronaVac (18).

### 3.3. Efficacy of COVID-19 Vaccines against Delta Variant for Preventing Severe Infection or Death

**Figure 4:**
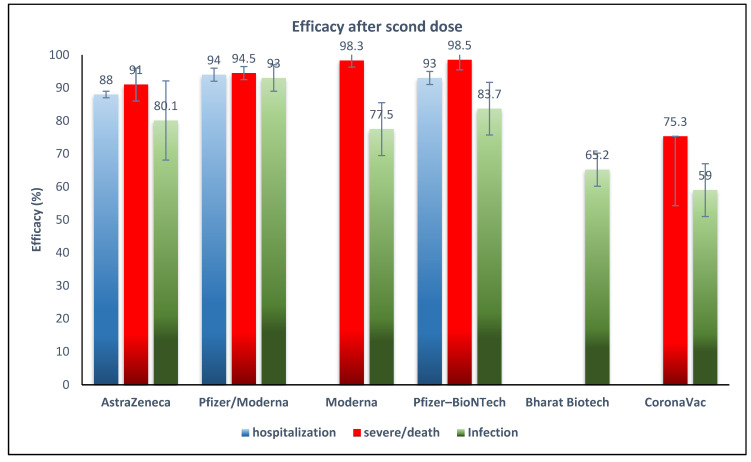
Effectiveness of COVID-19 vaccines after the second dose against Delta variant infection, hospitalization, and severe infection or death. * Reported for mRNA vaccines (Pfizer/Moderna).

Pfizer-BioNTech had the highest VE of 0.985 (95% CI 0.95–0.99) against the Delta variant for preventing severe infection or death. Subsequently, Moderna had a VE of 0.983 (95% CI 0.936–0.957), AstraZeneca had a VE of 0.91 (95% CI 0.88–0.92), and CoronaVac had a VE of 0.753 (95% CI 0.71–0.79) against the Delta variant for preventing severe infection or death. All reports were after the second dose of vaccines (Figure 4).

### 3.4. Efficacy of COVID-19 Vaccines against Delta Variant Hospitalization 

Pfizer-BioNTech had a VE of 0.93 (95% CI 0.88–0.96) [36] and AstraZeneca had a VE of 0.88 (95% CI 0.86–0.896) against the prevalence of hospitalization from the Delta variant [30].

## 4. Discussion

Several vaccines have been authorized for SARS-CoV-2, and the development of new vaccines is ongoing [37,38,39]. However, the recurrent appearance of new variants of SARS-CoV-2 has raised concern regarding the VE against all variants. The Delta variant with increased transmissibility and more severe infections first appeared in India and replaced the previously circulating variants, even in areas considered to have high vaccination rates for eligible individuals 18 years and older [29]. An increased incidence of SARS-CoV-2 infection at the time of the Delta variant’s predominance has been suspected to be associated with the decreased effectiveness of vaccines against Delta [40,41,42,43,44].

The Delta variant (B.1.617.2), similar to other new variants of SARS-CoV-2, has developed multiple mutations primarily in the S protein, such as L452R. The L452R mutation, which is in the receptor-binding domain of the S1 subunit, can prevent the binding of the neutralizing antibodies to the virus and decrease the efficacy of vaccine-induced antibodies [45]. The L452R and T478K mutations have been associated with increased transmissibility of the Delta variant [46].

To evaluate the pooled VE against the Delta variant, this study comprehensively analyzed the available data from fifteen studies on five COVID-19 vaccines and showed 53.8% overall VE after the first dose that increased to a level of 81.9% after the second dose. The large difference between overall VE after the first and second doses supports having a full vaccination course for optimal VE. Since studies included in our meta-analysis were observational, except for one clinical trial, we were able to compare our results with a recent meta-analysis of real-world studies. The authors of that study reported a pooled VE for the prevention of SARS-CoV-2 of 41% after the first dose and 85% after the second dose. They also reported the pooled VE for the prevention of SARS-CoV-2 VOCs, which was 74% for the Delta variant, the highest for the Alpha variant at 85%, 75% for the Beta variant, and the lowest for the Gamma variant at 54% [39]. A similar finding of lower pooled VE against Delta, Beta, and Gamma compared to Alpha was reported by other studies [47,48]. It was also noted that higher VE against Alpha compared to Delta was only present in preventing mild infection, but when the endpoint was severe COVID-19, the efficacy was the same [47].

According to our results, the highest VE occurred after a full vaccination course, with two mRNA-based vaccines having a VE of 83.7% (Pfizer/BioNTech) and 77.5% (Moderna), and the vector vaccine AstraZeneca having a VE of 80%. According to the WHO Target Product Profiles for COVID-19 Vaccines, a “Clear demonstration of efficacy (on population basis) ideally with ~50% point estimate” has been recommended as a minimum measure for VE [49], which shows that all five vaccines in this study have acceptable VE to prevent the SARS-CoV-2 Delta variant. The high VE reported after the first dose of Moderna (72%) may be important to decrease the risk of infection during the time between doses. Although the two inactivated virus-based vaccines showed lower VE, in light of the high infectivity and severity of infection with Delta, even a VE of 65% for Bharat Biotech and 59% for CoronaVac in our results would decrease the disease burden.

Reports on other types of vaccines were scarce, which necessitates an urgent need to study the effectiveness of other vaccines against the Delta variant. Only one study reported the VE after a third dose, which was about 97% for Pfizer-BioNTech and Moderna and 64% for CoronaVac, with higher titers of neutralizing antibodies compared to the primary doses [18]. With regard to VE against Delta-associated complications including severe infection or death, this study shows that Pfizer-BioNTech, Moderna, and AstraZeneca have about 90% VE after the second dose. VE against hospitalization was also high for Pfizer-BioNTech (93%) and AstraZeneca (88%). A meta-analysis study of phase II/III clinical trials before the emergence of the Delta variant reported an overall 95% vaccine efficacy for mRNA-based vaccines, higher than that reported in our study, and they reported 80% vaccine efficacy for viral vector vaccines, equal to this study [50]. Another report supported the higher VE of the mRNA vaccines against Variants of Concern, including Delta [48].

In addition to supporting the acceptable effectiveness of COVID-19 vaccines against the Delta variant, this study also shows different levels of VE, from 83.7% for Pfizer/BioNTech to 59% for CoronaVac. The difference may have partially originated from the different numbers of cases that were included for each vaccine. However, responsiveness to vaccines depends on multiple host and environmental factors such as age, gender, co-morbidities, and season, in addition to vaccination factors and the time after vaccination [51]. For example, the Pfizer-BioNTech vaccine, which had 95% efficacy in the primary multinational clinical trial [52], was reported to have a 64% and 90% VE after seven days of the second dose in two Danish populations, a group of residents of long-term care facilities and a group of healthcare workers, respectively [52]. Such information leads one to appreciate that there are multiple factors including side effect risks of various vaccine formulations in addition to a given VE.

Although long-term VE is not in the scope of this study, the waning of long-term VE may explain part of the breakthrough infections and increased incidence of COVID-19 at the time of Delta’s predominance. It has been reported that after 6–8 months, the effectiveness of Moderna and Pfizer-BioNTech decreased, but a third dose markedly increased the efficacy against the Delta variant [18]. A retrospective cohort study reported that the VE for Pfizer-BioNTech was high for both Delta and non-Delta variants, but it decreased after the 4–5-month follow-up. However, the VE for this vaccine against hospitalization was high for Delta and non-Delta variants up to six months [36].

The population vaccination rate is another challenge associated with the COVID-19 waves after the emergence of the new variants. A computational model of VE necessary for the elimination of the COVID-19 pandemic showed that with a VE of 80% when the reproduction number (R_0_) of the virus is 2.5, the population vaccination rate should be 60%. With the same VE, when the R_0_ increases to 3.5, the vaccination rate should be 75% at a minimum [53]. It is plausible that for the Delta variant with a reproduction number range of 3.2 to 8 [54], the population vaccination rate has to significantly exceed 75%. Population vaccination rates to date are just beginning to include children, which affects overall herd immunity, and the ongoing transmission of the virus in all unvaccinated cohorts.

At the time of revising the present manuscript (December 2021), a new VOC, B.1.1.529, named Omicron, emerged. This variant has mutations at the S1–S2 furin cleavage site and mutations in the receptor-binding domain, which appear to facilitate some degree of immune escape [55]. Early anecdotal data suggest mRNA-based vaccines are around 30–40% effective at preventing infections and 70% effective at preventing severe disease. This may be due to the timing of the onset where most people are approaching 6 months since their second dose, leading to waning immunity. This is now influencing governments towards recommending a third booster dose to control this hypervirulent VOC.

## 5. Limitations

There are potential limitations in this study. First, except for one clinical trial, all the included studies were observational including various population characteristics, which warrants cautious interpretation of the results. Second, there was a limitation in the number of published studies. Third, there was a limitation in the duration of follow-ups after vaccine doses. Fourth, there were differences amongst the included populations in the various studies regarding case numbers, ethnic and geographical regions, vaccine population coverage rates, etc. Fifth, under-reporting of individuals infected with the Delta variant, either vaccinated or not, particularly when health systems are overrun, including the availability and ability to test and monitor COVID-19 infection rates or sequence for variant identification, has the potential to skew the data in observational studies. Additionally, severe cases are more likely to come to medical attention and thus be documented and captured, which is critical for monitoring severe outcomes related to COVID-19 and for monitoring vaccine effectiveness. Finally, there are statistical limitations due to various study biases, particularly publication bias and heterogeneity. We conducted subgroup analyses to detect the sources of heterogeneity. The subgroup analysis delineated some study characteristics including different vaccine doses and different COVID-19 complications such as infection, hospitalization, severe illness, and death. Other subgroup and sensitivity analyses were conducted; however, only significant results are reported. For the prevention of language bias, we did not have any language limitations in this study. For the prevention of publication bias, we searched various websites and databases such as the WHO and CDC to find gray literature. However, bias and heterogeneity are unavoidable in systematic reviews and meta-analysis studies. Therefore, all of these limitations should be considered when interpreting the outcomes.

## 6. Conclusions

The present study supports the short-term effectiveness of the Pfizer-BioNTech, Moderna, AstraZeneca, Bharat Biotech, and CoronaVac vaccines for the prevention of infection and the reduction in the severity of illness and hospitalizations associated with the Delta variant. Ongoing monitoring of protective immunity and administration of booster doses in a reasonable timeframe are recommended. Prior to the emergence of new variants, implementation of practical strategies for improving the vaccination rate is urgently required, including vaccinating children, and carefully monitoring the timeline of waning immunity. 

## Figures and Tables

**Table 1:** Characteristics of included publications.

Study ID	Country	Name	Company	Study Type	Vaccine Type	Dose	Day *	Efficacy Against (%)			Ref
								Infection	Hospitalization	Severe/Death	
Bernal et al.	England	ChAdOx1 nCoV-19	AstraZeneca	case– control	Adeno vector	1	21	30.00	NA	NA	[25]
Nasreen et al.	Canada	ChAdOx1 nCoV-19	AstraZeneca	case– control	Adeno vector	1	14	67.00	NA	NA	[25]
Pouwels et al.	England	ChAdOx1 nCoV-19	AstraZeneca	case– control	Adeno vector	1	21	46.00	NA	NA	[26]
Xiao-Ning Li	China	CoronaVac	Sinovac Biotech	case– control	Inactivated	1	14	13.80	NA	NA	[27]
Nasreen et al.	Canada	mRNA-1273	Moderna	case– control	mRNA	1	14	72.00	NA	NA	[25]
Ben Y. Reis	NA	BNT162b2	Pfizer- BioNTech	case– control	mRNA	1	14–20	59.00	NA	NA	[28]
Ben Y. Reis	NA	BNT162b2	Pfizer- BioNTech	case– control	mRNA	1	21–27	66.00	NA	NA	[28]
Bernal et al.	England	BNT162b2	Pfizer- BioNTech	case– control	mRNA	1	21–56	35.60	NA	NA	[25]
Nasreen et al.	Canada	BNT162b2	Pfizer- BioNTech	case– control	mRNA	1	14	56.00	NA	NA	[25]
Pouwels et al.	England	BNT162b2	Pfizer- BioNTech	case– control	mRNA	1	21	57.00	NA	NA	[26]
Bernal et al.	England	ChAdOx1 nCoV-19	AstraZeneca	case– control	Adeno vector	2	14	67	NA	NA	[25]
Pouwels et al.	England	AdOx1 nCoV-19	AstraZeneca	case– control	Adeno vector	2	14	71	NA	NA	[26]
Pouwels et al.	England	ChAdOx1 nCoV-19	AstraZeneca	case– control	Adeno vector	2	>14	67	NA	NA	[26]
Sheikh et al.	Scotland	AdOx1 nCoV-19	AstraZeneca	cohort	Adeno vector	2	>14	91	NA	91	[29]
Paul M McKeigue	Scotland	AdOx1 nCoV-19	AstraZeneca	case– control	Live	2	>14	91	88	NA	[30]
Raches Ella	India	BBV152	Bharat Biotech	clinical trial III	Inactivated	2	14	65.2	NA	NA	[31]
xiao-Ning Li	China	CoronaVac	Sinovac Biotech	case– control	Inactivated	2	14	59	NA	NA	[27]
Nasreen et al.	Canada	mRNA-1273	Moderna	case– control	mRNA	2	14	72	NA	NA	[25]
Russell S	NA	mRNA-1273	Moderna	case– control	mRNA	2	28	74	NA	NA	[32]
Tenforde	NA	mRNA-1273	Moderna	case– control	mRNA	2	91–168	86	NA	NA	[33]
Paul M McKeigue	Scotland	BNT162b2/mRNA-1273	Pfizer/Moderna **	case– control	mRNA	2	>14	92	91	NA	[30]
Po Ying Chia	Singapore	BNT162b2/mRNA-1273	Pfizer/ Moderna *	cohort	mRNA	2	>14	96	NA	NA	[34]
Lindsay T. Keegan	USA	BNT162b2/mRNA-1273/Ad26.COV2.S	Pfizer/ Moderna/ J&J ***		mRNA/ adeno vector	2	>14	82	NA	NA	[35]
Russell S	NA	BNT162b2	Pfizer- BioNTech	case– control	mRNA	2	28	73	NA	NA	[32]
Tenforde	NA	BNT162b2	Pfizer- BioNTech	case– control	mRNA	2	14–84	86	NA	NA	[33]
Ben Y. Reis	NA	BNT162b2	Pfizer- BioNTech	case– control	mRNA	2	7–21	88	NA	NA	[28]
Sara Y. Tartof	USA	BNT162b2	Pfizer- BioNTech	cohort	mRNA	2	7	93	93	NA	[36]
Sara Y. Tartof	USA	BNT162b2	Pfizer- BioNTech	cohort	mRNA	2	120	53	NA	NA	[36]
Bernal et al.	England	BNT162b2	Pfizer- BioNTech	case– control	mRNA	2	14	88	NA	NA	[25]
Nasreen et al.	Canada	BNT162b2	Pfizer- BioNTech	case– control	mRNA	2	7	87	NA	NA	[25]
Pouwels et al.	England	BNT162b2	Pfizer- BioNTech	case– control	mRNA	2	0–13	82	NA	NA	[26]
Pouwels et al.	England	BNT162b2	Pfizer- BioNTech	case– control	mRNA	2	>14	80	NA	NA	[26]
Sheikh et al.	Scotland	BNT162b2	Pfizer- BioNTech	cohort	mRNA	2	>14	90.00	NA	90.00	[29]
Xinhua Chen	NA	mRNA-1273	Moderna	cohort	mRNA	3	14–30	97.00	NA	98.30	[18]
Xinhua Chen	NA	BNT162b2	Pfizer- BioNTech	cohort	mRNA	3	14–30	97.20	NA	98.90	[18]
Xinhua Chen	NA	CoronaVac	Sinovac Biotech	cohort	Inactivated	3	60	63.80	NA	75.30	[18]

ChAdOx1: chimpanzee (Ch) adenovirus-vectored vaccine; BNT162b2: BioNTech; J&J: Johnson & Johnson; Whole-Virion Inactivated SARS-CoV-2 Vaccine (BBV152) NA: not available. * Reported vaccine efficacy after vaccination; ** reported for mRNA vaccines (Pfizer/Moderna); *** total of 52.9% with Pfizer, 38.1% with Moderna, and 9.05% with Janssen (Pfizer/Moderna/J&J).

**Table 2:** Effectiveness of COVID-19 vaccines against Delta variant.

Dose	Group	Number of Studies	Vaccine Effectiveness	95% Interval		Test of Null (2-Tailed)		Heterogeneity			
				Lower Limit	Upper Limit	Z-Value	*p*-Value	Q-Value	df (Q)	*p*-Value	I-Squared
After First Dose	AstraZeneca	3	**0.439**	0.301	0.588	−0.802	0.422	135.8	2.0	<0.001	98.5
	CoronaVac	1	**0.138**	0.076	0.237						
	Moderna	1	**0.720**	0.589	0.822						
	Pfizer-BioNTech	5	**0.567**	0.520	0.613	2.810	0.005	1011.3	4.0	<0.001	99.6
	Overall	10	**0.538**	0.496	0.579	1.794	0.073	5515.7	9.0	<0.001	99.8
After Second Dose	Pfizer/Moderna *	2	**0.939**	0.884	0.969	7.6	<0.001	4.3	1.0	<0.001	77.0
	Pfizer-BioNTech	10	**0.837**	0.672	0.928	3.5	<0.001	359,532.1	9.0	<0.001	100.0
	Pfizer/Moderna/ J&J **	1	**0.820**	0.819	0.821						
	AstraZeneca	5	**0.801**	0.705	0.872	5.2	<0.001	7502.6	4.0	<0.001	99.9
	Moderna	3	**0.775**	0.673	0.852	4.7	<0.001	3.1	2.0	0.2	35.9
	Bharat Biotech	1	**0.652**	0.642	0.662						
	CoronaVac	1	**0.590**	0.475	0.696						
	Overall	23	**0.819**	0.818	0.819	690.6	<0.001	453,257.3	22.0	<0.001	100.0
After Third Dose	Moderna	1	**0.970**	0.964	0.978						
	Pfizer-BioNTech	1	**0.972**	0.960	0.978						
	CoronaVac	1	**0.638**	0.631	0.643						

* Reported for mRNA vaccines (Pfizer/Moderna); ** total of 52.9% with Pfizer, 38.1% with Moderna, and 9.05% with Janssen (Pfizer/Moderna/J&J).

## Data Availability

All data needed to evaluate the conclusions in the paper are included and/or available within the Supplementary Materials. Additional data related to this paper may be requested from the authors.

## Supplementary Materials

The following supporting information can be downloaded at: https://www.mdpi.com/article/10.3390/vaccines10010023/s1. Table S1. Search strategy. Table S2. Quality assessment of included studies.
