# Supplementary material for: Effectiveness of COVID-19 Vaccines against Delta (B.1.617.2) Variant: A Systematic Review and Meta-Analysis of Clinical Studies
Source: Vaccines (Basel). 2021 Dec 25;10(1):23. doi: 10.3390/vaccines10010023 (PMC8778641; doi:10.3390/vaccines10010023)
Supplement: Supplementary file 1 [file vaccines-10-00023-s001.zip › vaccines-1488198-supplementary.pdf]

Supplementary material

Effectiveness of COVID-19 Vaccines Against Delta (B.1.617.2) Variant: A Systematic Review and Meta-Analysis of Clinical Studies.

Ali Pormohammad[1], Mohammad Zarei[2,3], Saied Ghorbani[4], Mehdi Mohammadi[5], Saeideh Aghayari Sheikh Neshin[6], Alireza Khatami [7], Diana L. Turner[8], Shirin Djalalinia[9], Seied Asadollah Mousavi[10], Heydar Ali Mardani-Fard[11], Amir Kasaeian[12*], Raymond J. Turner[13*]

1. Department of Biological Sciences, University of Calgary, Calgary, AB, Canada ali.pormohammad@ucalgary.ca
2. Renal Division, Brigham & Women's Hospital, Harvard Medical School, Boston, Massachusetts, USA. mzarei@hsph.harvard.edu
3. John B. Little Center for Radiation Sciences, Harvard T.H. Chan School of Public Health, Boston, MA 02115. mzarei@hsph.harvard.edu
4. Department of Virology, Faculty of Medicine, Iran University of Medical Science, Tehran, Iran. vet.s.ghorbani@gmail.com
5. Department of Biological Sciences, University of Calgary, Calgary, AB, Canada mehdi.mohammadiashan@ucalgary.ca
6. Neuroscience Research Center, Guilan University of Medical Sciences, Rasht, Iran saeidehyari88@gmail.com.
7. Department of Virology, Faculty of Medicine, Iran University of Medical Science, Tehran, Iran Email: akh.alirezakhatami@gmail.com
8. Department of Family Medicine, Cumming School of Medicine, University of Calgary, Calgary, AB T2N 4N1, Canada
9. Non-Communicable Diseases Research Center, Endocrinology and Metabolism Population Sciences Institute, Tehran University of Medical Sciences, Tehran, Iran
10. Hematology, Oncology and Stem Cell Transplantation Research Center, Research Institute for Oncology, Hematology and Cell Therapy, Tehran University of Medical Sciences, Tehran, Iran.
11. Department of Mathematics, Yasouj University, Yasouj, IRAN h_mardanifard@yahoo.com
12. Research Institute for Oncology, Hematology and Cell Therapy, Shariati Hospital, Tehran University of Medical Sciences, Kargar Shomali Street, Tehran, 1411713131, Iran
13. Department of Biological Sciences, University of Calgary, Calgary, AB, Canada turnerr@ucalgary.ca

*Corresponding Authors:

Amir Kasaeian, Research Institute for Oncology, Hematology and Cell Therapy, Shariati Hospital, Tehran University of Medical Sciences, Kargar Shomali Street, Tehran, 1411713131, Iran

Raymond J. Turner, Professor, Department of Biological Sciences, University of Calgary
Phone No: +1 (403) 220-4308 Mail: turnerr@ucalgary.ca

Predicting vaccine efficacy over time across different vaccines and clinical endpoints

In the included studies, fallowing method used for prediction vaccine efficacy.

We used the model to predict vaccine protection against prototype strain based on the relationship between neutralizing antibody level and vaccine efficacy (equation 1);

we used an integral based on normal distribution of neutralization level to calculate the probability of being protected (equation 2).

$$E_I\ (n|n_{50}, k) = \frac{1}{1+e^{-k(n-n_{50})}}, (1)$$

$$P\ (n_{50}, k, \mu_s, \sigma_s) = \int_{-\infty}^{+\infty} \mathrm{E}_I\ (n|n_{50}, k)\ f(n|\mu_s, \sigma_s) dn, (2)$$

A logistic model was used to model the relationship for equation (1), where EI is the vaccine efficacy given the log-transformed neutralizing antibody titer n, and n50 is the neutralization titer at which an individual will have a 50% protective efficacy. The parameter k controls the steepness of the logistic function. The logistic relationship for different clinical endpoints was constructed by changing the n50 and k.4 For equation (2), assuming that neutralizing antibodies follow a normal distribution with mean μs and standard deviation σs, f indicates the probability density function of neutralization titer, and P represents the proportion of vaccinated population for a study s that will be protected. To enhance comparability between different studies with different neutralization assays, the neutralization titer (μs) was normalized in each study with the means of titers in corresponding convalescent individuals reported in Phase I/II trials against prototype strains. Confidence intervals of predicted efficacy against the prototype strain were estimated by using the Hessian H and standard error (s.e. = sqrt(diag(H-1))); 95% CIs were calculated as ±1.96*s.e. of the estimated parameters. For predictions of vaccine efficacy against the Delta variant, we added log-transformed n-fold-reduction of the Delta variant on the neutralizing antibody level into equation (3), and predicted variant-specific efficacy by using equations (1) and (2).

$$\mu_s^v\ = \mu_s + \bar{F}^v, (3)$$

Where Fv is the mean log-transformed n-fold-change (vaccine-specific) in neutralization titer against the Delta variant, μs is the normalized neutralization titer (vaccine-specific) against the prototype strain, and μv s is the normalized neutralization titer (vaccine-specific) for the Delta variant. Confidence intervals of predicted efficacy against the Delta variant were calculated by imputing the 95% confidence intervals of the n-fold changes of neutralization titers.

Table S1. Search strategy.

ID	ID Search Terms	Results
**Medline (Pubmed)**		
**#1**	Delta variant and vaccine	**270**
**#2**	b.1.617.2 variant and vaccine	**115**
**#3**	covid-19 delta variant and vaccine	**231**
**#4**	Sars-cov-2 delta variant and vaccine	**925**
**Science Direct**		
**#1**	Delta variant and vaccine	**6226**
**#2**	b.1.617.2 variant and vaccine	**241**
**#3**	covid-19 delta variant and vaccine	**831**
**#4**	Sars-cov-2 delta variant and vaccine	**842**
**Web of sciences**		
**#1**	Delta variant and vaccine	**187**
**#2**	b.1.617.2 variant and vaccine	**103**
**#3**	covid-19 delta variant and vaccine	**156**
**#4**	Sars-cov-2 delta variant and vaccine	**654**
**EMBASE**		

**#1**	Delta variant and vaccine	**164**
**#2**	b.1.617.2 variant and vaccine	**98**
**#3**	covid-19 delta variant and vaccine	**143**
**#4**	Sars-cov-2 delta variant and vaccine	**432**
	**Scopus**	
**#1**	Delta variant and vaccine	**653**
**#2**	b.1.617.2 variant and vaccine	**231**
**#3**	covid-19 delta variant and vaccine	**457**
**#4**	Sars-cov-2 delta variant and vaccine	**1253**

Table S2. Quality assessment of included studies

Authors	Q1	Q2	Q3	Q4	Q5	Q6	Q7	Q8	Q9	Total QA score
Bernal	Yes	Yes	Yes	Yes	Yes	Yes	Yes	Yes	Yes	9
Pouwels	Yes	Yes	Yes	Yes	Yes	Yes	Yes	Yes	Yes	9
Sheikh	Yes	Yes	Yes	yes	Yes	Yes	Yes	Yes	Yes	9
McKeigue	Yes	Yes	Yes	Yes	Yes	Yes	Yes	Yes	Yes	9
Raches	Yes	Yes	Yes	Yes	Yes	Yes	Yes	Yes	Yes	9
Xiao	Yes	Yes	no	Yes	Yes	Yes	Yes	Yes	Yes	8
Nasreen	Yes	Yes	no	Yes	Yes	Yes	Yes	Yes	Yes	8
Xinhua	yes	Yes	yes	Yes	Yes	Yes	Yes	Yes	Yes	9
Russell	Yes	Yes	Yes	Yes	Yes	Yes	Yes	Yes	Yes	9
Tenforde	Yes	Yes	no	Yes	Yes	Yes	Yes	Yes	Yes	8
Chia	Yes	Yes	Yes	yes	Yes	Yes	Yes	Yes	Yes	9
Keegan	Yes	Yes	Yes	Yes	Yes	Yes	Yes	Yes	Yes	9
Chen	yes	Yes	yes	Yes	Yes	Yes	Yes	Yes	Yes	9
Reis	Yes	Yes	Yes	Yes	Yes	Yes	Yes	Yes	Yes	9
Tartof	yes	Yes	yes	Yes	Yes	Yes	Yes	Yes	Yes	9

Q1= Was the sample frame appropriate to address the target population?
Q2= Were study participants sampled in an appropriate way?
Q3= Was the sample size adequate?
Q4= Were the study subjects and the setting described in detail?
Q5= Was the data analysis conducted with sufficient coverage of the identified sample?
Q6= Were valid methods used for the identification of the condition?
Q7= Was the condition measured in a standard, reliable way for all participants?
Q8= Was there appropriate statistical analysis?
Q9= Was the response rate adequate, and if not, was the low response rate managed appropriately?
